# How Effective Is Radiotherapy in the Ultrasonographic Structural Characteristics of the Submandibular Glands?

**DOI:** 10.5152/eurasianjmed.2024.23227

**Published:** 2024-06-01

**Authors:** Gözde Açikgöz, Hayati Murat Akgül, Orhan Sezen, Hilal Kiziltunç Özmen

**Affiliations:** 1 Department of Oral and Maxillofacial Radiology, Pamukkale University Faculty of Dentistry, Denizli, Türkiye; 2 Department of Radiation Oncology, Atatürk University Faculty of Medicine, Erzurum, Türkiye; 3 Anesthesiology Clinical Research Office, Atatürk University Faculty of Medicine, Erzurum, Türkiye

**Keywords:** Head and neck cancer, radiotherapy, submandibular gland, ultrasonography

## Abstract

**Background:**

Radiotherapy affects salivary glands more intensely than it does other organs, and salivary gland dysfunction can continue during or after treatment. The aim of this study was to examine structural alterations in submandibular glands through ultrasonography following head-neck radiotherapy in patients and to evaluate the impact of radiation dose on these modifications.

**Methods:**

Forty-six submandibular glands were assessed ultrasonographically for the changes in echogenicity, echotexture, and margin and the influence of the radiation dose on these changes before radiotherapy at 3 time points: the second and sixth months following starting treatment. Statistical analysis of the data was performed using a chi-square test.

**Results:**

Significant relationship in 3 ultrasonographic structural characteristics—echogenicity, echotexture, and margin— of submandibular glands (*P* < .001, *P* < .001, and *P* < .001, respectively) were observed before and at the second and sixth months after radiotherapy. There was found a significant correlation between the radiation dose groups in the change of echotexture at 2 different time periods after radiotherapy (*P* < .001, *P* < .05, respectively) and in the change of margin at the second month after radiotherapy onset (*P* < .05).

**Conclusion:**

Preceding radiotherapy, submandibular glands typically exhibited hyperechoic echogenicity, homogeneous ecotextures, and regular margins. However, after radiotherapy, there was an observable transformation characterized by isoechoic/hypoechoic features, heterogeneous textures, and irregular margins. With the passage of time following radiotherapy, there was a tendency for the parenchyma structure to gradually revert to a normal state. Also, the radiation dose generally has an effect on the structural changes of the submandibular glands.

**Main Points**

- Submandibular glands, like parotid glands, are also severely affected by radiation.
- Ultrasonography may be provided with small benefit in the evaluation of salivary glands post radiotherapy.
- Head–neck radiotherapy causes structural changes in the submandibular glands.
- These structural changes tend to return to normal again after radiotherapy.
- Only on the echotexture, significant changes were observed in the radiation dose groups.

## Introduction

Head and neck cancers rank sixth after lung, breast, uterus, prostate, and colorectal cancers among the most common worldwide and they account for approximately 3%-5% of all cancer types.^1,2^ While the incidence of these cancers is approximately 3 to 4 times higher in males,^3,4^ their distribution according to age also varies and is more common between the ages of 50 to 70.^5,6^

Surgery, chemotherapy, radiotherapy methods, and combinations thereof are widely implemented in the treatment of head and neck cancers. Although radiotherapy is used effectively in their treatment, it causes serious local complications. Radiotherapy has serious oral complications such as salivary gland dysfunction, dry mouth, oral mucositis, and osteonecrosis.^2,4^ These complications may cause disruption and interruption of cancer treatment, an increase in treatment costs, and deterioration in the patient’s quality of life.

Salivary glands are highly sensitive to radiotherapy.^7^ Head and neck radiotherapy causes structural, dimensional, physiological, and functional alterations in the salivary glands, and salivary gland dysfunction continues during or after treatment.^7^ Different methods, such as questionnaires, histological evaluation, sialometry, conventional radiography, sialography, scintigraphy, computed tomography (CT), positron emission tomography (PET), magnetic resonance imaging, and ultrasonography (USG), have been reported in the literature for the accurate evaluation of these effect on salivary glands.^2,7-17^ While evaluating the dimensional and structural changes in the salivary glands, although successful results can be achieved with advanced imaging methods, such as MR and CT, researchers investigated methods aimed at removing drawbacks, including high radiation dose and the difficulty of application. In recent years, USG and the Doppler technique, which are more comfortable, accessible, non-ionizing, non-invasive, and economical compared to other imaging methods, have been accepted as the first step in the evaluation of salivary glands after radiotherapy.^2,9,11,12,14,18-22^

All parts of the submandibular glands can be visualized by USG, and the normal submandibular gland is homogeneous and slightly hyperechoic on USG when compared to adjacent muscles.^9,18,20^ The structure of the salivary glands exposed to radiotherapy is different from the normal gland structure,^21^ and can be safely evaluated with USG.^2^ Yang et al^12,13^ observed that healthy parotid glands have soft tissue echogenicity and homogeneous parenchymal structure, while parotid glands after radiotherapy are distinctly heterogeneous with hypoechoic areas and hyperechoic lines/spots. Dost and Kaiser,^22^ the healthy salivary gland echotexture was observed homogeneously, while Jindal et al^11^ and Cheng et al^8^ found that the echotexture of salivary glands after radiotherapy varied from homogeneous to heterogenous. In general, salivary gland margins are also observed to change from regular to irregular after radiotherapy.^2,9,11,12,14^ Studies on this subject have mostly focused on the parotid gland.^2,11,14-16,19^ Whereas, as well as the parotid glands, the submandibular glands are seriously affected by radiation. Additionally, considering the width of USG probes, it should be noted that submandibular glands can be examined with ease and comfort than parotid glands.

The purpose of our research was to compare 3 ultrasonographic structural characteristics of submandibular glands before and at 2 time periods after starting head and neck radiotherapy and to assess how the changes are influenced by the radiation dose.

## Material and Methods

### Study Planning

This research was designed to evaluate, by USG, the structural changes of the submandibular glands before radiotherapy (stage 1), and at the end of the second (stage 2) and sixth (stage 3) months after starting radiotherapy in patients who will receive head–neck radiotherapy at the Faculty of Medicine, Department of Radiation Oncology at Atatürk University from October 2017 to December 2018. It was planned that the patients would be examined clinically and radiologically at every stage.

### Ethics Committee Approval

After planning, the study received approval from the “Atatürk University Faculty of Dentistry Ethics Committee” (Decision number #2017/11/65, Date: 21.09.2017). and was carried out in accordance with the Helsinki Declaration of 1975, as revised in 2008. Patients were given detailed information about the study, and written and oral informed consent was subsequently obtained from them.

### Patients

The study enrolled a total of 23 people (21 males and 2 females, with a mean age of 54.3 ± 14.63 ± 14.6 years) who received radiotherapy in the head and neck region. Fourteen (60.9%) of the 23 patients diagnosed with head and neck cancer were for laryngeal cancer, 4 (17.4%) for lymphoma, and 1 (4.3%) each for nasopharynx cancer, hypopharynx cancer, maxillary sinus cancer, mucoepidermoid carcinoma in parotid gland, and lower lip cancer. In the selection of the patients to be included in the study, the criteria of having no history of previous head and neck radiotherapy, not having previous chemotherapy, not having had a systemic disease affecting the salivary glands, and not having had a previous operation in the submandibular gland region were taken into consideration.

Detailed anamnesis was obtained from the patients. Patients’ clinical and radiographic examinations were carried out to identify dental problems and improve oral hygiene at all stages. Additionally, the submandibular glands were investigated using USG at 3 time periods (stage 1, 2, 3) to determine whether there was any change in these glands.

### Radiotherapy

The patients’ radiotherapy was planned and applied by the Faculty of Medicine, Department of Radiation Oncology at Atatürk University. Computed tomography scans were taken from all patients, ensuring proper immobilization through the use of a thermoplastic mask. In the 3D conformal planning system, CT images were combined with PET-CT and/or MR images of the patients. Target volumes and organs at risk were contoured and the most convenient radiotherapy areas for each patient were completed with the Eclipse program. Patients approved for treatment underwent radiotherapy using the Varian Trilogy Version 13.6 Linear Accelerator (Varian Medical Systems, USA) device, employing Intensity-modulated radiation therapy (IMRT) and/or the Volumetric modulated arc therapy (VMAT) technique, along with Image-guided radiation therapy (IGRT). The patients’ treatment protocol was a daily dose ranging from 1.6 Gy to 3 Gy, administered over 13 to 36 fractions at 6 MV energy every weekday. The total radiation doses ranged from 30.6 Gy to 75 Gy (mean=61.2 ± 13 Gy). These treatments were exclusively radiation-based and were not combined with chemotherapy. Submandibular glands received radiation doses ranging from 0.2 Gy to 72.5 Gy (mean = 37.8 ± 23.1 Gy), categorized into 3 groups:^4^ ≤30 Gy (n = 17), <30 to ≤60 Gy (n = 19), and >60 Gy (n = 10).

### Ultrasonographic Examination

The study encompassed a total of 46 submandibular glands, representing 23 patients and examined in terms of echogenicity, echotexture, and margins before radiotherapy (stage 1), and in the second (stage 2) and sixth (stage 3) months following starting radiotherapy,^2,4,10,11^ by the Toshiba Aplio 300 USG (Toshiba Medical Systems Corporation, Tokyo, Japan) at the Atatürk University Faculty of Dentistry Department of Oral and Maxillofacial Radiology. To ensure standardization, the patients were positioned with their sagittal plane perpendicular to the floor and their occlusal plane parallel to the floor. Bilateral USG evaluation was conducted extraorally using a 12-MHz linear transducer. The probe was placed in 2 perpendicular planes: parallel to the lower edge of the mandible and vertical to its body. All USG images were evaluated during this examination, and results were recorded immediately by a blind dentomaxillofacial radiologist.

**Figure 1.**
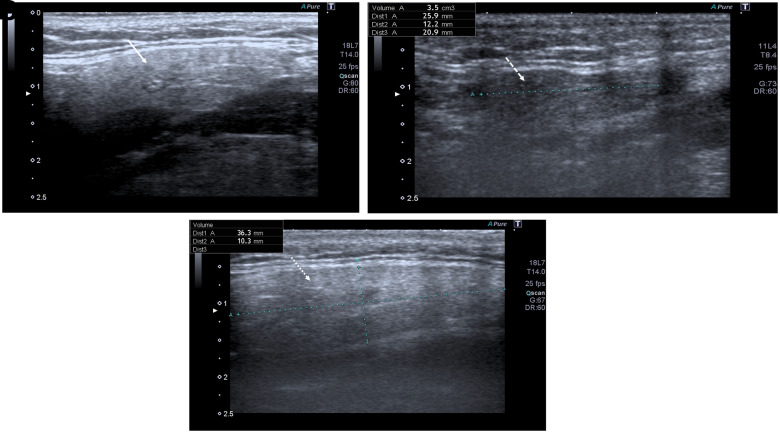
Evaluation of the parenchyma of the submandibular glands of a nasopharynx cancer patient, in terms of echogenicity. A) Submandibular gland with hyperechoic echogenicity (straight arrow) before radiotherapy (stage 1). B) Submandibular gland with hypoechoic echogenicity (long dashed arrow) at the second month (stage 2) after starting radiotherapy. C) Submandibular gland with isoechoic echogenicity (short dashed arrow) at the sixth month (stage 3) after starting radiotherapy.

**Figure 2.**
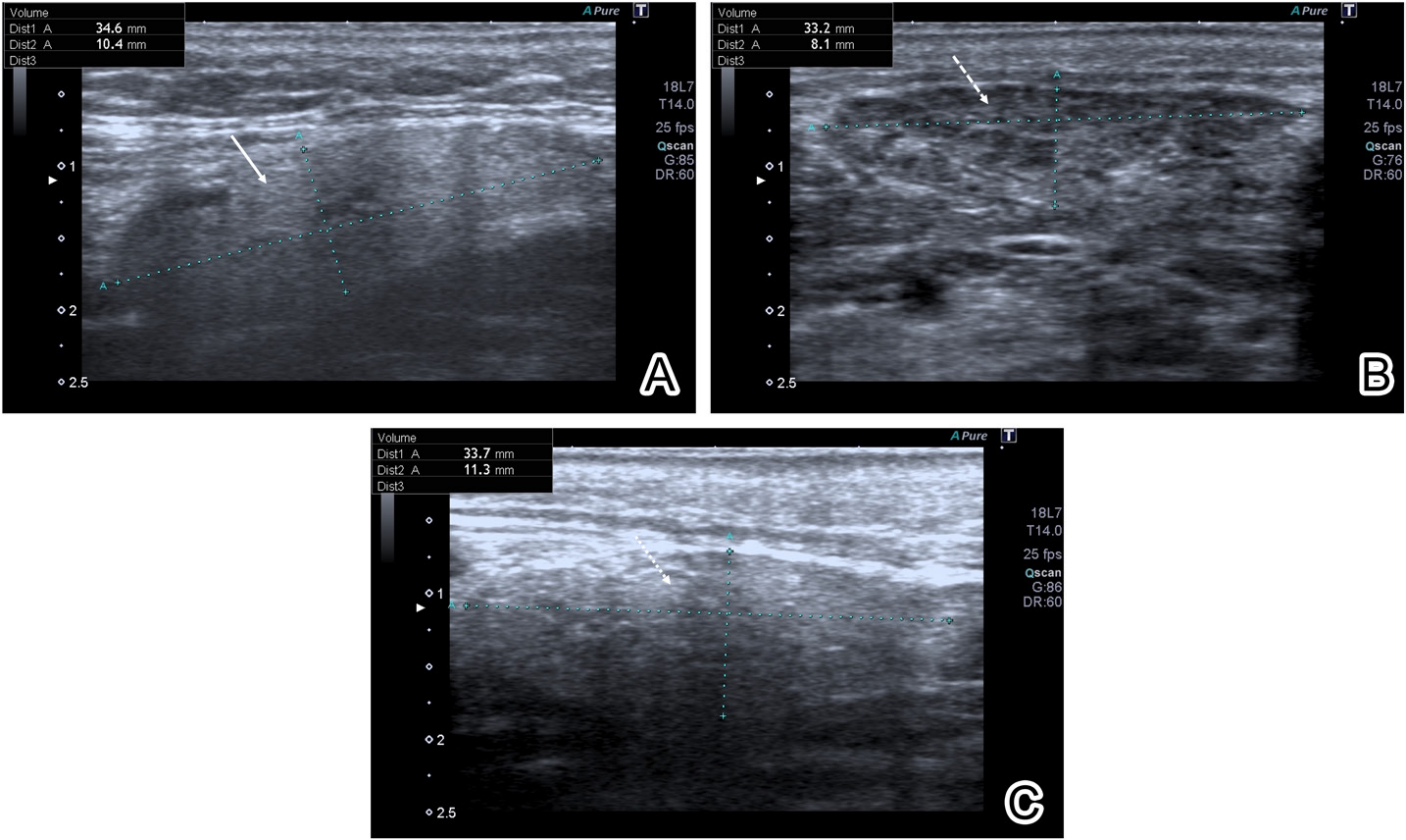
Evaluation of the parenchyma of the submandibular glands of a nasopharynx cancer patient, in terms of echotexture. A) Submandibular gland with homogeneous echotexture (straight arrow) before radiotherapy (stage 1). B) Submandibular gland with heterogeneous echotexture (long dashed arrow) at the second month (stage 2) after starting radiotherapy. C) Submandibular gland with homogeneous echotexture (short dashed arrow) at the sixth month (stage 3) after starting radiotherapy.

**Figure 3.**
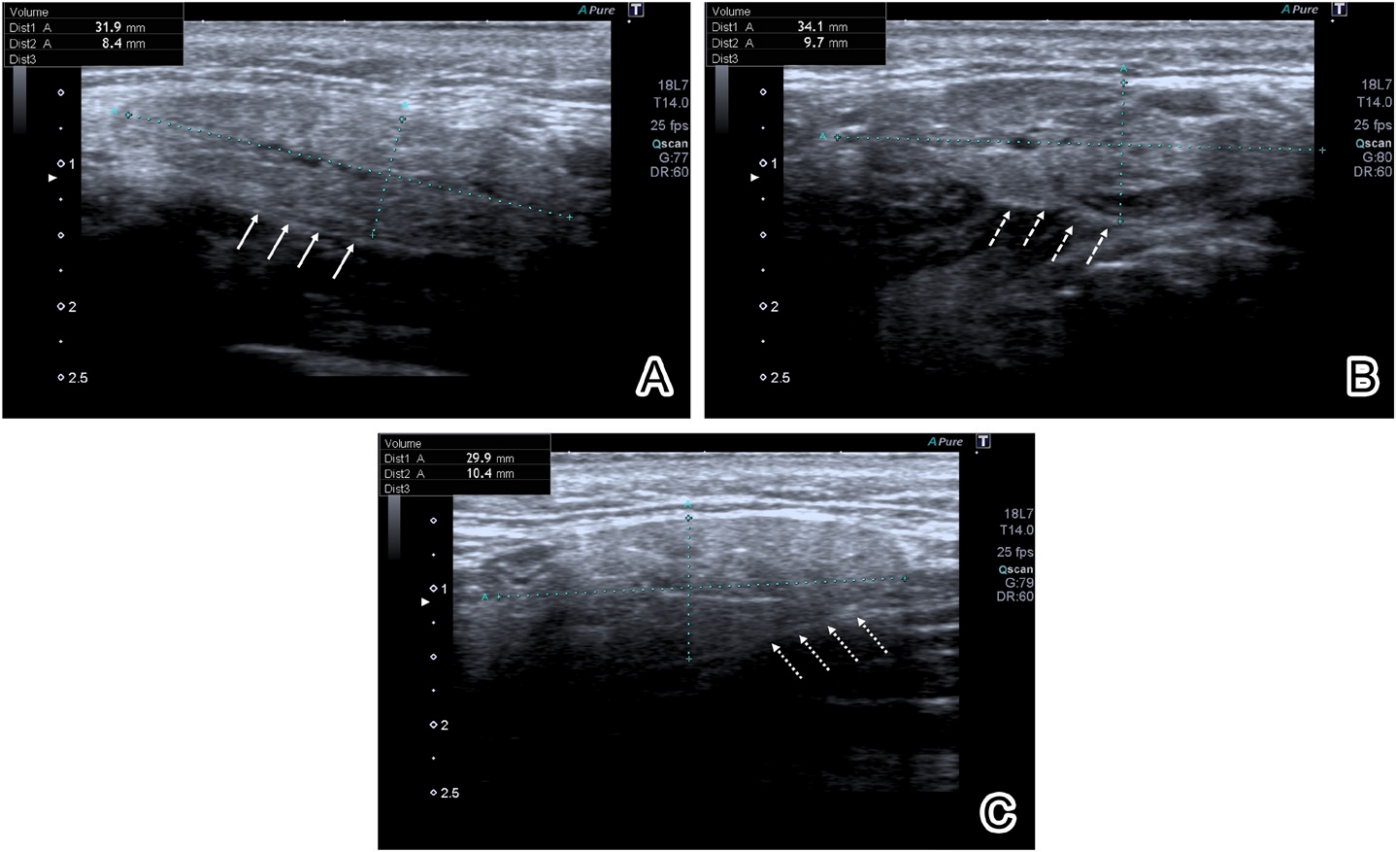
Evaluation of the margins of the submandibular glands of a nasopharynx cancer patient. A) Submandibular gland with a regular margin (straight arrow) before radiotherapy (stage 1). B) Submandibular gland with an irregular margin (long dashed arrow) at the second month (stage 2) after starting radiotherapy. C) Submandibular gland with a regular margin (short dashed arrow) at the sixth month (stage 3) after starting radiotherapy.

The parenchyma of the submandibular glands was evaluated in terms of echogenicity as hyperechoic, isoechoic, and hypoechoic.^2,11,19^ The echogenicity of the glands was compared with the echogenicity of the adjacent muscle.^2,8,18,19^ The evaluation criteria for parenchyma structure included categorizing brighter echogenicity than adjacent soft tissues as hyperechoic, the same echogenicity as isoechoic, and darker echogenicity as hypoechoic (as shown in Figure 1). The parenchyma of the submandibular glands was evaluated as homogeneous and heterogeneous in terms of echotexture (as shown in Figure 2), and their margins were also evaluated as regular and irregular (as shown in Figure 3).^2,11,19^

### Statistical Analysis

All data was analyzed statistically using the SPSS ver. 20.0 (IBM SPSS Corp.; Armonk, NY, USA). To compare the echogenicity, echotexture, and margin of the submandibular glands at 3 different time periods (stage 1, 2, 3), a chi-square test was employed. Also, the same parameters at the second (stage 2) and the sixth (stage 3) month following starting radiotherapy were analyzed by a chi-square test according to the radiation doses of submandibular glands. A relationship between the groups was deemed statistically significant when the *P*-value was below .05.

## Results

Table 1 shows the change in echogenicity, echotexture, and margins of the submandibular glands before radiotherapy (stage 1) and at the second (stage 2) and sixth (stage 3) months following starting radiotherapy. During the 6-month period, it was observed that the echogenicity returns to baseline USG findings in approximately one-third of the glands. Additionally, a return to before treatment was observed in approximately half of the glands during this period in the echotexture and margins. Significant statistical relationships were observed among the groups in these 3 parameters (*P* < .001).

Table 2 and 3 show comparisons of echogenicity, echotexture, and margins according to the radiation dose of submandibular glands. There was not found a statistically significant relationship between the groups for echogenicity at the second (stage 2) and sixth (stage 3) month following starting radiotherapy. In both time periods, the homogeneity rate was observed to be lower at high radiation doses (>60 Gy) and there was found a statistically significant relationship between the groups (*P* < .001, *P* < .05, respectively). Additionally, gland margins were observed less regularly at high radiation doses (>60 Gy) in both time periods, while there was found a statistically significant relationship between the groups only in the second (stage II) month (*P* < .05).

## Discussion

The findings of our study expose that, after radiotherapy, submandibular gland echogenicity transforms from hyperechoic to iso-hypoechoic, echotextures change from homogeneous to heterogenous, and margins from regular to irregular. The heterogeneous appearance in the gland parenchyma is attributed to hyperechoic lines/spots and hypoechoic regions, indicative of fibrosis and inflammatory response.^11,12,22,23^ Additionally, as a result of acinar atrophy occurring in the glands, it becomes difficult to distinguish the gland from neighboring soft tissues.^2,8,11,23^ However in our study, during the time period of 6 months, it was observed that the echogenicity returns to baseline USG findings in approximately one-third of the glands, and that the echotexture and margin of the glands also return to baseline in about half of the glands. The parenchyma of the salivary glands is very sensitive to radiation. Therefore, these structural changes are an inevitable result when administering the total radiation doses to our patients.

In a study conducted with the use of USG by Johari et al,^16^ it was observed that the echogenicity of both parotid and submandibular glands changed as hypoechoic and irregular in their margins at 6 to 7 weeks after radiotherapy (*P* < .001). Similarly, Ying et al^14^ defined the parenchyma of the parotid glands after radiotherapy as hypoechoic or isoechoic and heterogeneous. In the study conducted with long-term follow-up by Wu et al,^23^ the echogenicity of the parotid gland was evaluated by USG at 6, 12, 18, and 24 months after radiotherapy. While all parotid glands were hyperechoic before radiotherapy, it was observed that echogenicity changed to hypoechoic or isoechoic 6 months after radiotherapy. Also, significant differences were found between before radiotherapy and all time periods after radiotherapy, while no significant difference was found between time periods after radiotherapy. In the examination at different time periods (before radiotherapy, 2 weeks after radiotherapy, and 6 to 7 weeks after radiotherapy) by Imanimognaddam et al,^2^ the echogenicity of both parotid and submandibular glands before radiotherapy was shown as hyperechoic/isoechoic as compared with neighboring muscles, homogeneous of their parenchymas, and regular in their margins, while the structure of the glands was observed as hypoechoic, heterogeneous, and irregular in margins in the post-radiotherapy examinations. It was determined that these differences were statistically significant. In another study, although Cheng et al^19^ generally observed as hyperechoic the echogenicity of the healthy submandibular glands and hypoechoic in patients who underwent radiotherapy, they found no statistically significant difference between the 2 groups. While the healthy submandibular glands had 90% homogenous echotexture, 28% homogeneous echotexture was observed in patients with radiotherapy. In addition, Cheng et al^19^ observed that the submandibular glands in the control group generally had regular margins, and those of the patient group had irregular margins.

The intensity of complications related to radiotherapy varies based on factors like the therapy type, fraction dose, boundaries of the therapy region, and duration of therapy, particularly total radiation dose.^13,24^ Previous research has revealed that constant damage to the salivary glands occurred at total radiation doses of ≥24 Gy^25^ and ≥60 Gy.^26^ In the present study, no found statistical relationship was found between radiation dose groups for some parameters in the ultrasonographic structural characteristics of submandibular in both the second (stage 2) and sixth (stage 3) months, although some of the submandibular glands received a radiation dose of 60 Gy or more. If the population of patients in the groups was higher, it could be possible to emerge.

As a limitation of our research, long-term follow-up of patients (after 6months) and the correlation of USG results with other advanced imaging methods and clinical symptoms, such as the quantity of saliva or oral dryness, could not be evaluated due to a shortage of time. These follow-ups should be realized to define the changes and complications that may occur in a long time post-radiotherapy. Additionally, further studies can be planned to examine the relationship of structural changes with clinical symptoms and other advanced imaging methods. On the other hand, the population of the study was restricted because of both the shortage of time and the patient capacity of the oncology department.

As a result, we can say that head and neck radiotherapy causes structural—echogenicity, echotexture, and margin—changes in the submandibular glands at an early stage, and these changes tend to return to baseline again the longer the time after radiotherapy (6 months). Although some results were also obtained according to the radiation dose received by the submandibular glands in our research, a definite conclusion could not be reached due to the small patient population in the radiation dose groups. In the assessment of structural changes related to radiotherapy, the studies conducted so far have focused mostly on the effects of radiotherapy on the parotid glands. Nevertheless, the findings of our research indicate that the submandibular are also significantly impacted by radiation. In this context, submandibular glands also should be emphasized as much as possible in the protection of salivary glands in head and neck radiotherapy patients. Additionally, USG may provide a small benefit compared to other advanced imaging methods, considering its many advantages such as not containing ionizing radiation, being easily accessible and could be evaluated by different double-blind radiologists.

## Figures and Tables

**Table 1.** Statistical comparisons of Echogenicity, Echotexture, and Margins of the Submandibular Glands Before Radiotherapy (Stage 1) and at the Second (Stage 2) and the Sixth Month (Stage 3) After Starting Radiotherapy

	Stage						*X* ^2^	P*
	1		2		3			
	n	%	n	%	n	%		
**Echogenicity** Hyperechoic Isoechoic Hypoechoic	43 1 2	93.5 2.2 4.3	18 21 7	39.1 45.7 15.2	26 16 4	56.5 34.8 8.7	31.27	.000**
**Echotexture** Homogenous Heterogenous	42 4	91.3 8.7	16 30	34.8 65.2	30 16	65.2 34.8	31.86	.000**
**Margin** Regular Irregular	41 5	89.1 10.9	7 39	15.2 84.8	24 22	52.2 47.8	50.36	.000**

*Chi-square test.

**P < .001.

**Table 2.** Statistical Comparisons of Echogenicity, Echotexture, and Margins According to Radiation Dose of Submandibular Glands at the Second (Stage 2) Month After Starting Radiotherapy

	Radiation Dose Groups						*X* ^2^	P*
	≤30 Gy		>30 to ≤60 Gy		>60 Gy			
	n	%	n	%	n	%		
**Echogenicity** Hyperechoic Isoechoic Hypoechoic	10 7 0	58.8 41.2 0	6 8 5	31.6 42.1 26.3	2 6 2	20 60 20	7.75	.101
**Echotexture** Homogenous Heterogenous	12 5	70.6 29.4	2 17	10.5 89.5	2 8	20 80	15.50	.000**
**Margin** Regular Irregular	6 11	35.3 64.7	0 19	0 100	1 9	10 90	8.93	.011***

*Chi-square test.

**P < .001.

***P < .05.

**Table 3.** Statistical Comparisons of Echogenicity, Echotexture, and Margins According to Radiation Dose of Submandibular Gland. at the Sixth (Stage 3) Month After Starting Radiotherapy

	Radiation Dose Groups						*X* ^2^	P*
	≤30 Gy		>30 to ≤60 Gy		>60 Gy			
	n	%	n	%	n	%		
**Echogenicity** Hyperechoic Isoechoic Hypoechoic	9 8 0	52.9 47.1 0	12 6 1	63.2 31.6 5.3	5 2 3	50 20 30	8.64	.071
**Echotexture** Homogenous Heterogenous	15 2	88.2 11.8	11 8	57.9 42.1	4 6	40 60	7.22	.027**
**Margin** Regular Irregular	11 6	64.7 35.3	9 10	47.4 52.6	4 6	40 60	1.84	.399

*Chi-square test.

**P < .05.
